# Business process performance measurement: a structured literature review of indicators, measures and metrics

**DOI:** 10.1186/s40064-016-3498-1

**Published:** 2016-10-18

**Authors:** Amy Van Looy, Aygun Shafagatova

**Affiliations:** Faculty of Economics and Business Administration – Department of Business Informatics and Operations Management, Ghent University, Tweekerkenstraat 2, 9000 Ghent, Belgium

**Keywords:** Business process, Performance measurement, Indicator, Measure, Metric, Structured literature review, Systematic literature review

## Abstract

Measuring the performance of business processes has become a central issue in both academia and business, since organizations are challenged to achieve effective and efficient results. Applying performance measurement models to this purpose ensures alignment with a business strategy, which implies that the choice of performance indicators is organization-dependent. Nonetheless, such measurement models generally suffer from a lack of guidance regarding the performance indicators that exist and how they can be concretized in practice. To fill this gap, we conducted a structured literature review to find patterns or trends in the research on business process performance measurement. The study also documents an extended list of 140 process-related performance indicators in a systematic manner by further categorizing them into 11 performance perspectives in order to gain a holistic view. Managers and scholars can consult the provided list to choose the indicators that are of interest to them, considering each perspective. The structured literature review concludes with avenues for further research.

## Background

Since organizations endeavor to measure what they manage, performance measurement is a central issue in both the literature and in practice (Heckl and Moormann 2010; Neely 2005; Richard et al. 2009). Performance measurement is a multidisciplinary topic that is highly studied by both the management and information systems domains (business process management or BPM in particular). Different performance measurement models, systems and frameworks have been developed by academia and practitioners (Cross and Lynch 1988; Kaplan and Norton 1996, 2001; EFQM 2010; Kueng 2000; Neely et al. 2000). While measurement models were initially limited to financial performance (e.g., traditional controlling models), a more balanced and integrated approach was needed beginning in the 1990s due to the challenges of the rapidly changing society and technology; this approach resulted in multi-dimensional models. Perhaps the best known multi-dimensional performance measurement model is the Balanced Scorecard (BSC) developed by Kaplan and Norton (1996, 2001), which takes a four-dimensional approach to organizational performance: (1) financial perspective, (2) customer perspective, (3) internal business process perspective, and (4) “learning and growth” perspective. The BSC helps translate an organization’s strategy into operational performance indicators (also called performance measures or metrics) and objectives with targets for each of these performance perspectives. Even today, the BSC is by far the most used performance measurement approach in the business world (Bain Company 2015; Sullivan 2001; Ulfeder 2004).

Equally important for measuring an organization’s performance is process-oriented management or business process management (BPM), which is “about managing entire chains of events, activities and decisions that ultimately add value to the organization and its customers. These ‘chains of events, activities and decisions’ are called processes” (Dumas et al. 2013: p. 1). In particular, an organization can do more with its current resources by boosting the effectiveness and efficiency of its way of working (i.e., its business processes) (Sullivan 2001). In this regard, academic research also suggests a strong link between business process performance and organizational performance, either in the sense of a causal relationship (Melville et al. 2004; Smith and Reece 1999) or as distinctive indicators that co-exist, as in the BSC (Kaplan and Norton 1996, 2001).

Nonetheless, performance measurement models tend to give little guidance on how business (process) performance indicators can be chosen and operationalized (Shah et al. 2012). They are limited to mainly defining performance perspectives, possibly with some examples or steps to derive performance indicators (Neely et al. 2000), but without offering concrete indicators. Whereas fairly large bodies of research exist for both performance models and business processes, no structured literature review of (process) performance measurement has been carried out thus far. To the best of our knowledge, existing reviews cover one or another aspect of performance measurement; for instance, reviews on measurement models or evaluation criteria for performance indicators (Heckl and Moormann 2010; Neely 2005; Richard et al. 2009). Despite the considerable importance of a comprehensive and holistic approach to business (process) performance measurement, little is known regarding the state of the research on alternative performance indicators and their operationalization with respect to evaluating the performance of an organization’s work routines. To some extent, this lack of guidance can be explained by the fact that performance indicators are considered organization-dependent, given that strategic alignment is claimed by many measurement models such as the BSC (Kaplan and Norton 1996, 2001). Although the selection of appropriate performance indicators is challenging for practitioners due to the lack of best practices, it is also highly relevant for performance measurement.

The gap that we are studying is the identification and, in particular, the concretization/operationalization of process-related performance indicators. This study enhances the information systems literature, which focuses on the design and development of measurement systems without paying much attention to essential indicators. To fill this gap, our study presents a structured literature review in order to describe the current state of business process performance measurement and related performance indicators. The choice to focus on the business process management (BPM) discipline is motivated by the close link between organizational performance and business process performance, as well as to ensure a clear scope (specifically targeting an organization’s way of working). Accordingly, the study addresses the following research questions.

- RQ1. What is the current state of the research on business process performance measurement?
- RQ2. Which indicators, measures and metrics are used or mentioned in the current literature related to business process performance?

The objective of RQ1 is to identify patterns in the current body of knowledge and to note weaknesses, whereas RQ2 mainly intends to develop an extended list of measurable process performance indicators, categorized into recognized performance perspectives, which can be tailored to diverse purposes. This list could, for instance, serve as a supplement to existing performance measurement models. Practitioners can use the list as a source for best practice indicators from academic research to find and select a subset of performance indicators that fit their strategy. The study will thus not address the development of specific measurement systems but rather the indicators to be used within such systems. To make our intended list system-independent, we will begin with the BSC approach and extend its performance perspectives. Given this generic approach, the research findings can also be used by scholars when building and testing theoretical models in which process performance is one of the factors that must be concretized.

The remainder of this article is structured as follows. “Theoretical background” section describes the theoretical background of performance measurement models and performance indicators. Next, the methodology for our structured literature review is detailed in “Methods” section. The subsequent sections present the results for RQ1 (“Results for RQ1” section) and RQ2 (“Results for RQ2” section). The discussion of the results in provided in “Discussion” section, followed by concluding comments (“Conclusion” section).

## Theoretical background

This section addresses the concepts of performance measurement models and performance indicators separately in order to be able to differentiate them further in the study.

### Performance measurement models

According to overviews in the performance literature (Heckl and Moormann 2010; Neely 2005; Richard et al. 2009), some of the most cited performance measurement models are the Balanced Scorecard (Kaplan and Norton 1996, 2001), self-assessment excellence models such as the EFQM (2010), and the models by Cross and Lynch (1988), Kueng (2000) and Neely et al. (2000). A distinction should, however, be made between models focusing on the entire business (Kaplan and Norton 1996, 2001; EFQM 2010; Cross and Lynch 1988) and models focusing on a single business process (Kueng 2000; Neely et al. 2000).

#### Organizational performance measurement models

**Fig. 1:**
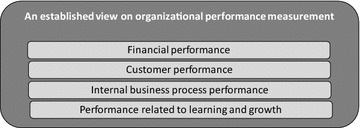
An overview of the performance perspectives in Kaplan and Norton (1996, 2001)

Organizational performance measurement models typically intend to provide a holistic view of an organization’s performance by considering different performance perspectives. As mentioned earlier, the BSC provides four perspectives for which objectives and performance indicators ensure alignment between strategies and operations (Fig. 1) (Kaplan and Norton 1996, 2001). Other organizational performance measurement models provide similar perspectives. For instance, Cross and Lynch (1988) offer a four-level performance pyramid: (1) a top level with a vision, (2) a second level with objectives per business unit in market and financial terms, (3) a third level with objectives per business operating system in terms of customer satisfaction, flexibility and productivity, and (4) a bottom level with operational objectives for quality, delivery, process time and costs. Another alternative view on organizational performance measurement is given in business excellence models, which focus on an evaluation through self-assessment rather than on strategic alignment, albeit by also offering performance perspectives. For instance, the EFQM (2010) distinguishes enablers [i.e., (1) leadership, (2) people, (3) strategy, (4) partnerships and resources, and (5) processes, products and services] from results [i.e., (1) people results, (2) customer results, (3) society results, and (4) key results], and a feedback loop for learning, creativity and innovation.

Since the BSC is the most used performance measurement model, we have chosen it as a reference model to illustrate the function of an organizational performance measurement model (Kaplan and Norton 1996, 2001). The BSC is designed to find a balance between financial and non-financial performance indicators, between the interests of internal and external stakeholders, and between presenting past performance and predicting future performance. The BSC encourages organizations to directly derive (strategic) long-term objectives from the overall strategy and to link them to (operational) short-term targets. Concrete performance measures or indicators should be defined to periodically measure the objectives. These indicators are located on one of the four performance perspectives in Fig. 1 (i.e., ideally with a maximum of five indicators per perspective).

Table 1 illustrates how an organizational strategy can be translated into operational terms using the BSC.

**Table 1:** An example of translating an organizational strategy into operational terms using the BSC

Perspective	Strategy	Objective	Indicator, measure or metric	Target			Initiative
				Year 1 (%)	Year 2 (%)	Year 3 (%)	
Customer	Operational excellence	Industry-leading customer loyalty	Customer satisfaction rating	80	85	90	Mystery shopper program Customer loyalty program

During periodical measurements using the BSC, managers can assign color-coded labels according to actual performance on short-term targets: (1) a green label if the organization has achieved the target, (2) an orange label if it is almost achieved, or (3) a red label if it is not achieved. Orange and red labels thus indicate areas for improvement.

Furthermore, the BSC assumes a causal or logical relationship between the four performance perspectives. An increase in the competences of employees (i.e., performance related to “learning and growth”) is expected to positively affect the quality of products and services (i.e., internal business process performance), which in turn will lead to improved customer perceptions (i.e., customer performance). The results for the previous perspectives will then contribute to financial performance to ultimately realize the organization’s strategy, mission and vision (Kaplan and Norton 1996, 2001). Hence, indicators belonging to the financial and customer perspectives are assumed to measure performance outcomes, whereas indicators from the perspectives of internal business processes and “learning and growth” are considered as typical performance drivers (Kaplan and Norton 2004).

Despite its widespread use and acceptance, the BSC is also criticized for appearing too general by managers who are challenged to adapt it to the culture of their organization (Butler et al. 1997) or find suitable indicators to capture the various aspects of their organization’s strategy (Shah et al. 2012; Vaivio 1999). Additionally, researchers question the choice of four distinct performance perspectives (i.e., which do not include perspectives related to inter-organizational performance or sustainability issues) (EFQM 2010; Hubbard 2009, Kueng 2000). Further, the causal relationship among the BSC perspectives has been questioned (Norreklit 2000). To some degree, Kaplan and Norton (2004) responded to this criticism by introducing strategy maps that focus more on the causal relationships and the alignment of intangible assets.

#### Business process performance measurement models

**Fig. 2:**
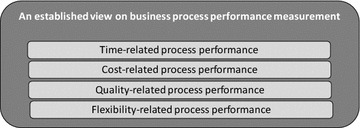
An overview of the performance perspectives in Dumas et al. (2013)

In addition to organizational models, performance measurement can also focus on a single business process, such as statistical process control, workflow-based monitoring or process performance measurement systems (Kueng 2000; Neely et al. 2000). The approach taken in business process performance measurement is generally less holistic than the BSC. For instance, in an established BPM handbook, Dumas et al. (2013) position time, cost, quality and flexibility as the typical performance perspectives of business process performance measurement (Fig. 2). Similar to organizational performance measurement, concrete performance measures or indicators should be defined for each process performance perspective. In this sense, the established perspectives of Dumas et al. (2013) seem to further refine the internal business process performance perspective of the BSC.

Neely et al. (2000), on the other hand, present ten steps to develop or define process performance indicators. The process performance measurement system of Kueng (2000) is also of high importance, which is visualized as a “goal and performance indicator tree” with five process performance perspectives: (1) financial view, (2) customer view, (3) employee view, (4) societal view, and (5) innovation view. Kueng (2000) thus suggests a more holistic approach towards process performance, similar to organizational performance, given the central role of business processes in an organization. He does so by focusing more on the different stakeholders involved in certain business processes.

### Performance indicators

Section “Performance measurement models” explained that performance measurement models typically distinguish different performance perspectives for which performance indicators should be further defined. We must, however, note that we consider performance measures, performance metrics and (key) performance indicators as synonyms (Dumas et al. 2013). For reasons of conciseness, this work will mainly refer to performance indicators without mentioning the synonyms. In addition to a name, each performance indicator should also have a concretization or operationalization that describes exactly how it is measured and that can result in a value to be compared against a target. For instance, regarding the example in Table 1, the qualitative statements to measure customer satisfaction constitute an operationalization. Nonetheless, different ways of operationalization can be applied to measure the same performance indicator. Since organizations can profit from reusing existing performance indicators and the related operationalization instead of inventing new ones (i.e., to facilitate benchmarking and save time), this work investigates which performance indicators are used or mentioned in the literature on business process performance and how they are operationalized.

Neely et al. (2000) and Richard et al. (2009) both present evaluation criteria for performance indicators (i.e., in the sense of desirable characteristics or review implications), which summarize the general consensus in the performance literature. First, the literature strongly agrees that performance indicators are organization-dependent and should be derived from an organization’s objectives, strategy, mission and vision. Secondly, consensus in the literature also exists regarding the need to combine financial and non-financial performance indicators. Nonetheless, disagreement still seems to exist in terms of whether objective and subjective indicators need to be combined, with objective indicators preferred by most advocates. Although subjective (or quasi-objective) indicators face challenges from bias, their use has some advantages; for instance, to include stakeholders in an assessment, to address latent constructs or to facilitate benchmarking when a fixed reference point is missing (Hubbard 2009; Richard et al. 2009). Moreover, empirical research has shown that subjective (or quasi-objective) indicators are more or less correlated with objective indicators, depending on the level of detail of the subjective question (Richard et al. 2009). For instance, a subjective question can be made more objective by using clear definitions or by selecting only well-informed respondents to reduce bias.

## Methods

We conducted a structured literature review (SLR) to find papers dealing with performance measurement in the business process literature. SLR can be defined as “a means of evaluating and interpreting all available research relevant to a particular research question, topic area, or phenomenon of interest” (Kitchenham 2007: p. vi). An SLR is a meta study that identifies and summarizes evidence from earlier research (King and He 2005) or a way to address a potentially large number of identified sources based on a strict protocol used to search and appraise the literature (Boellt and Cecez-Kecmanovic 2015). It is systematic in the sense of a systematic approach to finding relevant papers and a systematic way of classifying the papers. Hence, according to Boellt and Cecez-Kecmanovic (2015), SLR as a specific type of literature review can only be used when two conditions are met. First, the topic should be well-specified and closely formulated (i.e., limited to performance measurement in the context of business processes) to potentially identify all relevant literature based on inclusion and exclusion criteria. Secondly, the research questions should be answered by extracting and aggregating evidence from the identified literature based on a high-level summary or bibliometric-type of content analysis. Furthermore, King and He (2005) also refer to a statistical analysis of existing literature.

Informed by the established guidelines proposed by Kitchenham (2007), we undertook the review in distinct stages: (1) formulating the research questions and the search strategy, (2) filtering and extracting data based on inclusion and exclusion criteria, and (3) synthesizing the findings. The remainder of this section describes the details of each stage.

### Formulating the research questions and search strategy

A comprehensive and unbiased search is one of the fundamental factors that distinguish a systematic review from a traditional literature review (Kitchenham 2007). For this purpose, a systematic search begins with the identification of keywords and search terms that are derived from the research questions. Based on the research questions stipulated in the introduction, the SLR protocol (Boellt and Cecez-Kecmanovic 2015) for our study was defined, as shown in Table 2.

**Table 2:** The structured literature review protocol for this study, based on Boellt and Cecez-Kecmanovic (2015)

Protocol elements	Translation to this study
1/Research question	RQ1. What is the current state of the research on business process performance measurement? RQ2. Which indicators, measures and metrics are used or mentioned in the current literature related to business process performance?
2/Sources searched	Web of science database (until November 2015)
3/Search terms	Combining “business process*” and “performance indicator*”/“performance metric*”/“performance measur*”
4/Search strategy	Different search queries, with keywords in topic and title (Table 3)
5/Inclusion criteria	Include only papers containing a combination of search terms, defined in the search queries Include only papers indexed in the Web of Science from all periods until November 2015 Include only papers written in English
6/Exclusion criteria	Exclude unrelated papers, i.e., if they do not explicitly claim addressing the measurement of business process performance
7/Quality criteria	Only peer-reviewed papers are indexed in the web of science database

The ISI Web of Science (WoS) database was searched using predetermined search terms in November 2015. This database was selected because it is used by many universities and results in the most outstanding publications, thus increasing the quality of our findings. An important requirement was that the papers focus on “business process*” (BP). This keyword was used in combination with at least one of the following: (1) “performance indicator*”, (2) “performance metric*”, (3) “performance measur*”. All combinations of “keyword in topic” (TO) and “keyword in title” (TI) have been used.

 Table 3 shows the degree to which the initial sample sizes varied, with 433 resulting papers for the most permissive search query (TOxTO) and 19 papers for the most restrictive one (TIxTI). The next stage started with the most permissive search query in an effort to select and assess as many relevant publications as possible.

**Table 3:** The number of papers in the web of science per search query (until November 2015)

	(1) “Performance indicator*”	(2) “Performance metric*”	(3) “Performance measur*”	TOTAL
*Column keywords in TO*				
BP-TO	153	30	250	433
BP-TI	31	4	64	99
*Column keywords in TI*				
BP-TO	19	2	62	83
BP-TI	5	0	14	19

### Filtering and extracting data

**Fig. 3:**
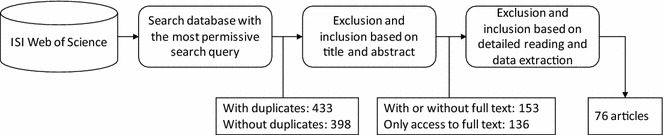
Exclusion of papers and number of primary studies

Figure 3 summarizes the procedure for searching and selecting the literature to be reviewed. The list of papers found in the previous stage was filtered by deleting 35 duplicates, and the remaining 398 papers were further narrowed to 153 papers by evaluating their title and abstract. After screening the body of the texts, 76 full-text papers were considered relevant for our scope and constituted the final sample (“Appendix 1”).

More specifically, studies were excluded if their main focus was not business process performance measurement or if they did not refer to indicators, measures or metrics for business performance. The inclusion of studies was not restricted to any specific type of intervention or outcome. The SLR thus included all types of research studies that were written in English and published up to and including November 2015. Furthermore, publication by peer-reviewed publication outlets (e.g., journals or conference proceedings) was considered as a quality criterion to ensure the academic level of the research papers.

### Synthesizing the findings

The analysis of the final sample was performed by means of narrative and descriptive analysis techniques. For RQ1, the 76 papers were analyzed on the basis of bibliometric data (e.g., publication type, publication year, geography) and general performance measurement issues by paying attention to the methodology and focus of the study. Details are provided in “Appendix 2”.

For RQ2, all the selected papers were screened to identify concrete performance indicators in order to generate a comprehensive list or checklist. The latter was done in different phases. In the first phase, the structured literature review allowed us to analyze which performance indicators are mainly used in the process literature and how they are concretized (e.g., in a question or mathematical formulation), resulting in an unstructured list of potential performance indicators. The indicators were also synthesized by combining similar indicators and rephrasing them into more generic terms.

The next phase was a comparative study to categorize the output of phase 1 into the commonly used measurement models in the performance literature (see “Theoretical background” section). For the purpose of this study, we specifically looked for those organizational performance models, mentioned in “Theoretical background” section, that are cited the most and that suggest categories, dimensions or performance perspectives that can be re-used (Kaplan and Norton 1996, 2001; EFQM 2010; Cross and Lynch 1988; Kueng 2000). Since the BSC (Kaplan and Norton 1996, 2001) is the most commonly used of these measurement models, we began with the BSC as the overall framework to categorize the observed indicators related to business (process) performance, supplemented with an established view on process performance from the process literature (Dumas et al. 2013). Subsequently, a structured list of potential performance indicators was obtained.

In the third and final phase, an evaluation study was performed to validate whether the output of phase 2 is sufficiently comprehensive according to other performance measurement models, i.e., not included in our sample and differing from the most commonly used performance measurement models. Therefore, we investigated the degree to which our structured list covers the items in two variants or concretizations of the BSC. Hence, a validation by other theoretical models is provided. We note that a validation by subject-matter experts is out of scope for a structured literature review but relates to an opportunity for further research.

## Results for RQ1

**Fig. 4:**
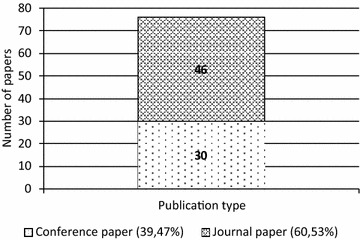
The distribution of the sampled papers per publication type (N = 76)

The final sample of 76 papers consists of 46 journal papers and 30 conference papers (Fig. 4), indicating a wide variety of outlets to reach the audience via operations and production-related journals in particular or in lower-ranked (Recker 2013) information systems journals.

**Fig. 5:**
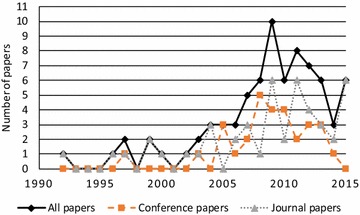
The chronological distribution of the sampled papers per publication type (N = 76)

When considering the chronological distribution of the sampled papers, Fig. 5 indicates an increase in the uptake of the topic in recent years, particularly for conference papers but also for journal publications since 2005.

**Fig. 6:**
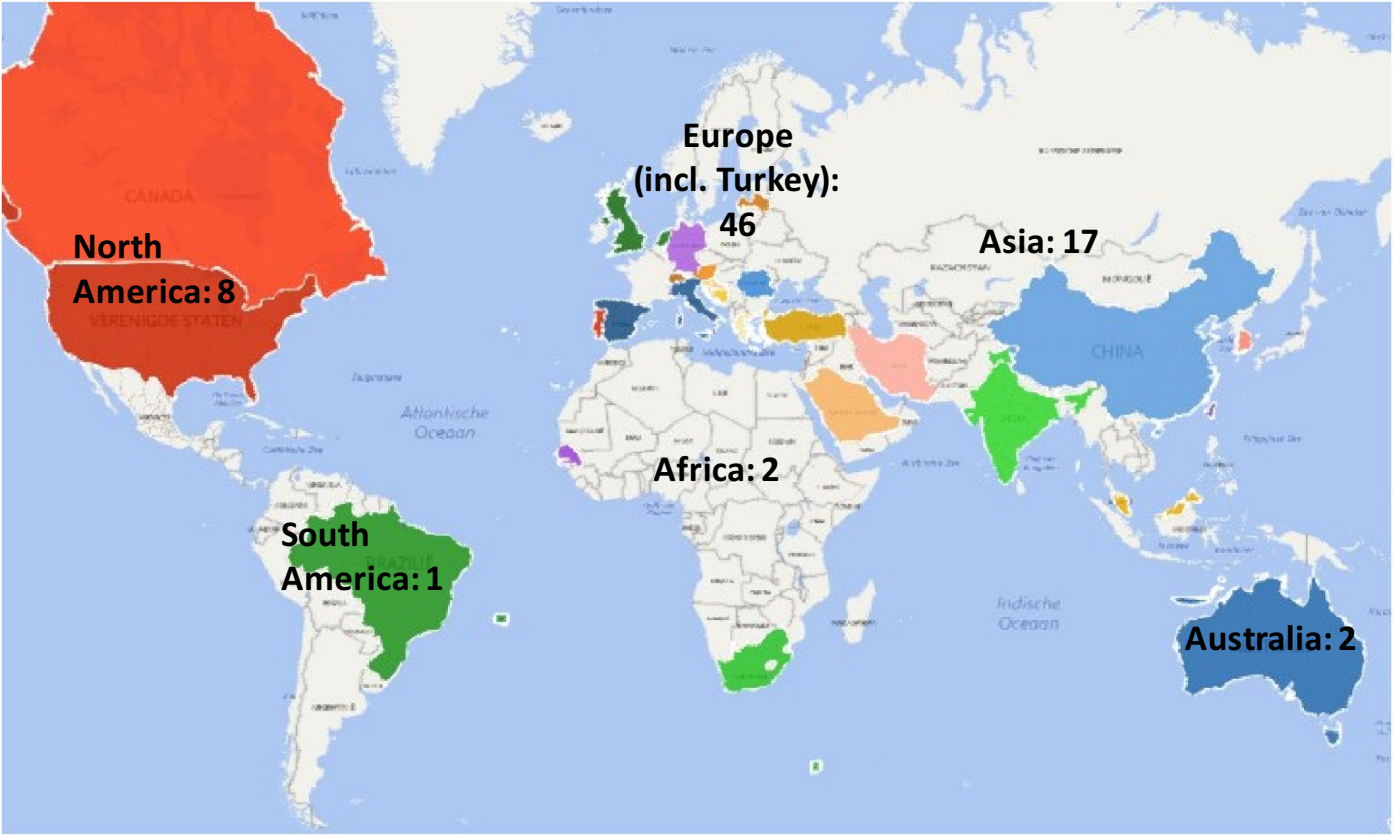
The geographical distribution of the sampled papers per continent, based on a paper’s first author (N = 76)

This uptake seems particularly situated in the Western world and Asia (Fig. 6). The countries with five or more papers in our sample are Germany (12 papers), the US (6 papers), Spain (5 papers), Croatia (5 papers) and China (5 papers). Figure 6 shows that business process performance measurement is a worldwide topic, with papers across the different continents. Nonetheless, a possible explanation for the higher coverage in the Western world could be due to its long tradition of measuring work (i.e., BSC origins).

**Fig. 7:**
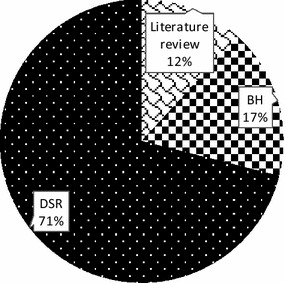
The distribution of the sampled journal papers per research paradigm (N = 76)

The vast majority of the sampled papers address artifacts related to business (process) performance measurement. When looking at the research paradigm in which the papers are situated (Fig. 7), 71 % address design-science research, whereas 17 % conduct research in behavioral science and 12 % present a literature review. This could be another explanation for the increasing uptake in the Western world, as many design-science researchers are from Europe or North America (March and Smith 1995; Peffers et al. 2012).

**Fig. 8:**
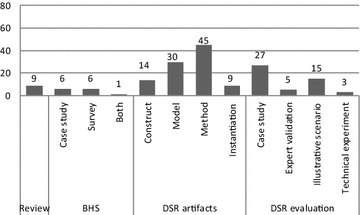
The distribution of the sampled journal papers per research method (N = 76)

Figure 8 supplements Fig. 7 by specifying the research methods used in the papers. For the behavioral-science papers, case studies and surveys are equally used. The 54 papers that are situated within the design-science paradigm explicitly refer to models, meta-models, frameworks, methods and/or tools. When mapping these 54 papers to the four artifact types of March and Smith (1995), the vast majority present (1) methods in the sense of steps to perform a task (e.g., algorithms or guidelines for performance measurement) and/or (2) models to describe solutions for the topic. The number of papers dealing with (3) constructs or a vocabulary and/or (4) instantiations or tools is much more limited, with 14 construct-related papers and 9 instantiations in our sample. We also looked at which evaluation methods, defined by Peffers et al. (2012), are typically used in the sampled design-science papers. While 7 of the 54 design-science papers do not seem to report on any evaluation effort, our sample confirms that most papers apply one or another evaluation method. Case studies and illustrative scenarios appear to be the most frequently used methods to evaluate design-science research on business (process) performance measurement.

The sampled design-science research papers typically build and test performance measurement frameworks, systems or models or suggest meta-models and generic templates to integrate performance indicators into the process models of an organization. Such papers can focus on the process level, organizational level or even cross-organizational level. Nonetheless, the indicators mentioned in those papers are illustrative rather than comprehensive. An all-inclusive list of generic performance indicators seems to be missing. Some authors propose a set of indicators, but those indicators are specific to a certain domain or sector instead of being generic. For instance, Table 4 shows that 36 of the 76 sampled papers are dedicated to a specific domain or sector, such as technology-related aspects or supply chain management.

**Table 4:** The number of sampled papers dedicated to a specific domain or sector (N = 76)

Domain or sector	Number of papers
IS/IT	7
Supply chain	5
Business network	3
Manufacturing	3
Services	3
Automobile	2
Banking/financial	2
Government	2
Health	2
Helpdesk/maintenance	2
Construction	1
HR	1
SME	1
Strategic planning	1
Telecom	1
Total	36

Furthermore, the reviewed literature was analyzed with regard to its (1) scope, (2) functionalities, (3) terminology, and (4) foundations.

Starting with scope, it is observed that nearly two-thirds of the sampled papers can be categorized as dealing with process-oriented performance measurement, whereas one-third focuses more on general performance measurement and management issues. Nonetheless, most of the studies of process performance also include general performance measurement as a supporting concept. A minor cluster of eight research papers specifically focuses on business process reengineering and measurement systems to evaluate the results of reengineering efforts. Furthermore, other researchers focus on the measurement and assessment of interoperability issues and supply chain management measurements.

Secondly, while analyzing the literature, two groups of papers were identified based on their functionalities: (1) focusing on performance measurement systems or frameworks, and (2) focusing on certain performance indicators and their categorization. Regarding the first group, it should be mentioned that while the process of building or developing a performance measurement system (PMS) or framework is well-researched, only a small number of papers explicitly address process performance measurement systems (PPMS). The papers in this first group typically suggest concrete steps or stages to be followed by particular organizations or discuss the conceptual characteristics and design of a performance measurement system. Regarding the second group of performance indicators, we can differentiate two sub-groups. Some authors focus on the process of defining performance indicators by listing requirements or quality characteristics that an indicator should meet. However, many more authors are interested in integrating performance indicators into the process models or the whole architecture of an organization, and they suggest concrete solutions to do so. Compared to the first group of papers, this second group deals more with the categorization of performance indicators into domains (financial/non-financial, lag/lead, external/internal, BSC dimensions) or levels (strategic, tactical, operational).

Thirdly, regarding terminology, different terms are used by different authors to discuss performance measurement. Performance “indicator” is the most commonly used term among the reviewed papers. For instance, it is frequently used in reference to a key performance indicator (KPI), a KPI area or a performance indicator (PI). The concept of a process performance indicator (PPI) is also used, mainly in the process-oriented literature. Performance “measure” is another prevalent term in the papers. The least-used term is performance “metric” (i.e., in only nine papers). Although the concepts of performance indicators, measures and metrics are used interchangeably throughout most of the papers, the concepts are sometimes defined in different ways. For instance, paper 17 defines a performance indicator as a metric, and paper 49 defines a performance measure as an indicator. On the other hand, paper 7 defines a performance indicator as a set of measures. Yet another perspective is taken in paper 74, which defines a performance measure as “a description of something that can be directly measured (e.g., number of reworks per day)”, while defining a performance indicator as “a description of something that is calculated from performance measures (e.g., percentage reworks per day per direct employee” (p. 386). Inconsistencies exist not only in defining indicators but also in describing performance goals. For instance, some authors include a sign (e.g., minus or plus) or a verb (e.g., decrease or increase) in front of an indicator. Other authors attempt to describe performance goals in a SMART way—for instance, by including a time indication (e.g., “within a certain period”) and/or target (e.g., “5 % of all orders”)—whereas most of the authors are less precise. Hence, a great degree of ambiguity exists in the formulation of performance objectives among to the reviewed papers.

**Fig. 9:**
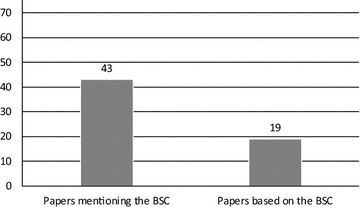
The importance of the BSC according to the sampled papers (N = 76)

Finally, regarding the papers’ foundations, “Performance measurement models” section already indicated that the BSC plays an important role in the general literature on performance management systems (PMS), while Kueng (2000) also offers influential arguments on process performance measurement systems (PPMS). In our literature review, we observed that the BSC was mentioned in 43 of the 76 papers and that the results of 19 papers were mainly based on the BSC (Fig. 9). This finding provides additional evidence that the BSC can be considered the most frequently used performance model in academia as well. However, the measurement model of Kueng (2000) was also mentioned in the sampled papers on PPMS, though less frequently (i.e., in six papers).

Interestingly, the BSC is also criticized by the sampled papers for not being comprehensive; for instance, due to the exclusion of environmental aspects, supply chain management aspects or cross-organizational processes. In response, some of the sampled papers also define sector-specific BSC indicators or suggest additional steps or indicators to make the process or business more sustainable (see Table 4). Nonetheless, the majority of the papers agree on the need for integrated and multidimensional measurement systems, such as the BSC, and on the importance of directly linking performance measurement to an organization’s strategy. However, while these papers mention the required link with strategy, the prioritization of indicators according to their strategic importance has been studied very little thus far.

## Results for RQ2

For RQ2, the sampled papers were reviewed to distinguish papers with performance indicators from papers without performance indicators. A further distinction was made between indicators found with operationalization (i.e., concretization by means of a question or formula) and those without operationalization. We note that for many indicators, no operationalization was available. We discovered that only 30 of the 76 sampled papers contained some type of performance indicator (namely 3, 5, 6, 7, 11, 16, 17, 18, 20, 22, 26, 27, 30, 35, 37, 40, 43, 46, 49, 51, 52, 53, 55, 57, 58, 59, 60, 66, 71, 73). In total, approximately 380 individual indicators were found throughout all the sampled papers (including duplicates), which were combined based on similarities and modified to use more generic terms. This resulted in 87 indicators with operationalization (“Appendix 3”) and 48 indicators without operationalization (“Appendix 4”).

The 87 indicators with operationalization were then categorized according to the four perspectives of the BSC (i.e., financial, customer, business processes, and “learning and growth”) (Kaplan and Norton 1996, 2001) and the four established dimensions of process performance (i.e., time, cost, quality, and flexibility) (Dumas et al. 2013). In particular, based in the identified indicators, we revealed 11 sub-perspectives within the initial BSC perspectives to better emphasize the focus of the indicators and the different target groups (Table 5): (1) financial performance for shareholders and top management, (2) customer-related performance, (3) supplier-related performance, (4) society-related performance, (5) general process performance, (6) time-related process performance, (7) cost-related process performance, (8) process performance related to internal quality, (9) flexibility-related process performance, (10) (digital) innovation performance, and (11) employee-related performance.

**Table 5:** A description of the observed performance perspectives, linked to the Balanced scorecard (Kaplan and Norton 1996, 2001)

Initial BSC perspectives	Observed perspectives based on target groups and focus	Scope of the performance indicators
1. Financial performance	1.1 Financial performance for shareholders and top management	Strategic financial data
2. Customer-related performance	2.1 Customer performance	Outcomes of external quality or meeting end user needs
	2.2 Supplier performance	External collaboration and process dependencies
	2.3 Society performance	Outcomes for other stakeholders and the environment during process work
3. Internal business process performance	3.1 General process performance	Descriptive data of process work, not related to time, costs, quality or flexibility
	3.2 Time-related process performance	Time-related data of process work
	3.3 Cost-related process performance	Operational financial data
	3.4 Process performance related to internal quality	Capability of meeting end user needs and internal user needs
	3.5 Flexibility-related process performance	Data of changes or variants in process work
4. Performance related to “learning and growth”	4.1 (Digital) innovation performance	Innovation of processes and innovation projects
	4.2 Employee performance	Staff contributions to process work and personal development

For reasons of objectivity, the observed performance indicators were assigned to a single perspective starting from recognized frameworks (Kaplan and Norton 1996, 2001; Dumas et al. 2013). Bias was further reduced by following the definitions of Table 5. Furthermore, the authors of this article first classified the indicators individually and then reached consensus to obtain a more objective categorization.

Additional rationale for the identification of 11 performance perspectives is presented in Table 6, which compares our observations with the perspectives adopted by the most commonly used performance measurement models (see “Theoretical background” section). This comparison allows us to highlight similarities and differences with other respected models. In particular, Table 6 shows that we did not observe a dedicated perspective for strategy (EFQM 2010) and that we did not differentiate between financial indicators and market indicators (Cross and Lynch 1988). Nonetheless, the similarities in Table 6 prevail. For instance, Cross and Lynch (1988) also acknowledge different process dimensions. Further, Kueng (2000) and the EFQM (2010) also differentiate employee performance from innovation performance, and they both add a separate perspective for results related to the entire society.

**Table 6:** The comparison of our observed performance perspectives with the perspectives taken in the most commonly used performance measurement models in the literature (Kaplan and Norton 1996, 2001; EFQM 2010; Kueng 2000; Cross and Lynch 1988)

Balanced scorecard (Kaplan and Norton 1996, 2001)	EFQM (2010)	Kueng (2000)	Cross and Lynch (1988)	Our observed performance perspectives
Financial perspective	Key results	Financial view	Financial measures Market measures	Financial performance for shareholders and top management
Customer perspective	Customer results	Customer view	Customer satisfaction	Customer performance Supplier performance Society performance
Internal business processes perspective	Enablers (processes/products/services, people, strategy, partnerships/resources, leadership)	Overall process performance based on the other views as driving forces	Flexibility Productivity Quality Delivery Process time Cost	General process performance Time-related process performance Cost-related process performance Process performance related to internal quality Flexibility-related process performance
“Learning and growth” perspective	People results Learning, creativity and innovation	Employee view Innovation view	–	(Digital) innovation performance Employee performance
–	Society results	Societal view	–	Society performance as a sub-perspective of customer performance (see above)

**Fig. 10:**
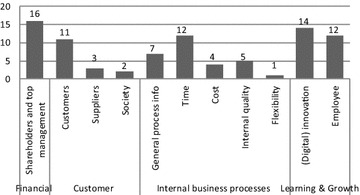
The number of performance indicators with operationalization per performance perspective

Figure 10 summarizes the number of performance indicators that we identified in the process literature per observed performance perspective. Not surprisingly, the initial BSC perspective of internal business process performance contains most of the performance indicators: 29 of 87 indicators. However, the other initial BSC perspectives are also covered by a relatively high number of indicators: 16 indicators for both financial performance and customer-related performance and 26 indicators for “learning and growth”. This result confirms the close link between process performance and organizational performance, as mentioned in the introduction.

A more detailed comparison of the perspectives provides interesting refinements to the state of the research. More specifically, Fig. 10 shows that five performance perspectives have more than ten indicators in the sample, indicating that academic research focuses more on financial performance for shareholders and top management and performance related to customers, process time, innovation and employees. On the other hand, fewer than five performance indicators were found in the sample for the perspectives related to suppliers, society, process costs and process flexibility, indicating that the literature focuses less on those perspectives. The latter remains largely overlooked by academic research, possibly due to the newly emerging character of these perspectives.

We must, however, note that the majority of the performance indicators are mentioned in only a few papers. For instance, 59 of the 87 indicators were cited in a single paper, whereas the remainder are mentioned in more than one paper. Eleven performance indicators are frequently mentioned in the process literature (i.e., by five or more papers). These indicators include four indicators of customer-related performance (i.e., customer complaints, perceived customer satisfaction, query time, and delivery reliability), three indicators of time-related process performance (i.e., process cycle time, sub-process turnaround time, and process waiting time), one cost-related performance indicator (i.e., process cost), two indicators of process performance related to internal quality (i.e., quality of internal outputs and deadline adherence), and one indicator of employee performance (i.e., perceived employee satisfaction).

Consistent with “Performance indicators” section, the different performance perspectives are a combination of financial or cost-related indicators with non-financial data. The latter also take the upper hand in our sample. Furthermore, the sample includes a combination of objective and subjective indicators, and the vast majority are objective indicators. Only eight indicators explicitly refer to qualitative scales; for instance, to measure the degree of satisfaction of the different stakeholder groups. For all the other performance indicators, a quantifiable alternative is provided.

It is important to remember that a distinction was made between the indicators with operationalization and those without operationalization. The list of 87 performance indicators, as given in “Appendix 3”, can thus be extended with those indicators for which operationalization is missing in the reviewed literature. Specifically, we found 48 additional performance indicators (“Appendix 4”) that mainly address supplier performance, process performance related to costs and flexibility, and the employee-related aspects of digital innovation. Consequently, this structured literature review uncovered a total of 135 performance indicators that are directly or indirectly linked to business process performance.

Finally, the total list of 135 performance indicators was evaluated for its comprehensiveness by comparing the identified indicators with other BSC variants that were not included in our sample. More specifically, based on a random search, we looked for two BSC variants in the Web of Science that did not fit the search strategy of this structured literature review: one that did not fit the search term of “business process*” (Hubbard 2009) and another that did not fit any of the performance-related search terms of “performance indicator*”, “performance metric*” or “performance measur*” (Bronzo et al. 2013). These two BSC variants cover 30 and 17 performance indicators, respectively, and are thus less comprehensive than the extended list presented in this study. Most of the performance indicators suggested by the two BSC variants are either directly covered in our findings or could be derived after recalculations. Only five performance indicators could not be linked to our list of 135 indicators, and these suggest possible refinements regarding (1) the growth potential of employees, (2) new markets, (3) the social performance of suppliers, (4) philanthropy, or (5) industry-specific events.

## Discussion

This structured literature review culminated in an extended list of 140 performance indicators: 87 indicators with operationalization, 48 indicators without operationalization and 5 refinements derived from two other BSC variants. The evaluation of our findings against two BSC variants validated our work in the sense that we present a more exhaustive list of performance indicators, with operationalization for most, and that only minor refinements could be added. However, the comprehensiveness of our findings can be claimed only to a certain extent given the limitations of our predefined search strategy and the lack of empirical validation by subject-matter experts or organizations. Notwithstanding these limitations, conclusions can be drawn from the large sample of 76 papers to respond to the research questions (RQs).

Regarding RQ1 on the state of the research on business process performance measurement, the literature review provided additional evidence for the omnipresence of the BSC. Most of the sampled papers mentioned or used the BSC as a starting point and basis for their research and analysis. The literature study also showed a variety of research topics, ranging from behavioral-science to design-science research and from a focus on performance measurement models to a focus on performance indicators. In addition to inconsistencies in the terminology used to describe performance indicators and targets, the main weakness uncovered in this literature review deals with the concretization of performance indicators supplementing performance measurement systems. The SLR results suggest that none of the reviewed papers offers a comprehensive measurement framework, specifically one that includes and extends the BSC perspectives, is process-driven and encompasses as many concrete performance indicators as possible. Such a comprehensive framework could be used as a checklist or a best practice for reference when defining specific performance indicators. Hence, the current literature review offers a first step towards such a comprehensive framework by means of an extended list of possible performance indicators bundled in 11 performance perspectives (RQ2).

Regarding RQ2 on process performance indicators, the literature study revealed that scholars measure performance in many different ways and without sharing much detail regarding the operationalization of the measurement instruments, which makes a comparison of research results more difficult. As such, the extended list of performance indicators is our main contribution and fills a gap in the literature by providing a detailed overview of performance indicators mentioned or used in the literature on business process performance. Another novel aspect is that we responded to the criticism of missing perspectives in the original BSC (EFQM 2010; Hubbard 2009; Kueng 2000) and identified the narrow view of performance typically taken in the process literature (Dumas et al. 2013). Figures 1 and 2 are now combined and extended in a more exhaustive way, namely by means of more perspectives than are offered by other attempts (Table 6), by explicitly differentiating between performance drivers (or lead indicators) and performance outcomes (or lag indicators), and by considering concrete performance indicators.

Our work also demonstrated that all perspectives in the BSC (Kaplan and Norton 1996, 2001) relate to business process performance to some degree. In other words, while the BSC is a strategic tool for organizational performance measurement, it is actually based on indicators that originate from business processes. More specifically, in addition to the perspective of internal business processes, the financial performance perspective typically refers to sales or revenues gained while doing business, particularly after executing business processes. The customer perspective relates to the implications of product or service delivery, specifically to the interactions throughout business processes, whereas the “learning and growth” perspective relates to innovations in the way of working (i.e., business processes) and the degree to which employees are prepared to conduct and innovate business processes. The BSC, however, does not present sub-perspectives and thus takes a more high-level view of performance. Hence, the BSC can be extended based on other categorizations made in the reviewed literature; for instance, related to internal/external, strategic/operational, financial/non-financial, or cost/time/quality/flexibility.

**Fig. 11:**
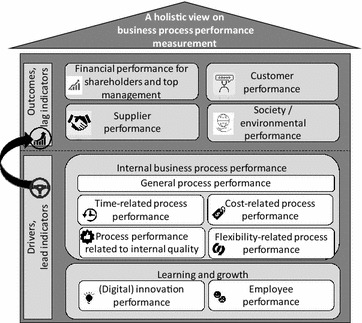
An overview of the observed performance perspectives in the business process literature

Therefore, this study refined the initial BSC perspectives into eleven performance perspectives (Fig. 11) by applying three other performance measurement models (Cross and Lynch 1988; EFQM 2010; Kueng 2000) and the respected Devil’s quadrangle for process performance (Dumas et al. 2013). Additionally, a more holistic view of business process performance can be obtained by measuring each performance perspective of Fig. 11 than can be achieved by using the established dimensions of time, cost, quality and flexibility as commonly proposed in the process literature (Dumas et al. 2013). As such, this study demonstrated a highly relevant synergy between the disciplines of process management, organization management and performance management.

We also found out that not all the performance perspectives in Fig. 11 are equally represented in the studied literature. In particular, the perspectives related to suppliers, society, process costs and process flexibility seem under-researched thus far.

The eleven performance perspectives (Fig. 11) can be used by organizations and scholars to measure the performance of business processes in a more holistic way, considering the implications for different target groups. For each perspective, performance indicators can be selected that fit particular needs. Thus, we do not assert that every indicator in the extended list of 140 performance indicators should always be measured, since “Theoretical background” section emphasized the need for organization-dependent indicators aligned with an organization’s strategy. Instead, our extended list can be a starting point for finding and using appropriate indicators for each performance perspective, without losing much time reflecting on possible indicators or ways to concretize those indicators. Similarly, the list can be used by scholars, since many studies in both the process literature and management literature intend to measure the performance outcomes of theoretical constructs or developed artifacts.

Consistent with the above, we acknowledge that the observed performance indicators originate from different models and paradigms or can be specific to certain processes or sectors. Since our intention is to provide an exhaustive list of indicators that can be applied to measure business process performance, the indicators are not necessarily fully compatible. Instead, our findings allow the recognition of the role of a business context (i.e., the peculiarities of a business activity, an organization or other circumstances). For instance, a manufacturing organization might choose different indicators from our list than a service or non-profit organization (e.g., manufacturing lead time versus friendliness, or carbon dioxide emission versus stakeholder satisfaction).

Another point of discussion is dedicated to the difference between the performance of specific processes (known as “process performance”) and the performance of the entire process portfolio (also called “BPM performance”). While some indicators in our extended list clearly go beyond a single process (e.g., competence-related indicators or employee absenteeism), it is our opinion that the actual performance of multiple processes can be aggregated to obtain BPM performance (e.g., the sum of process waiting times). This distinction between (actual) process performance and BPM performance is useful; for instance, for supplementing models that try to predict the (expected) performance based on capability development, such as process maturity models (e.g., CMMI) and BPM maturity models (Hammer 2007; McCormack and Johnson 2001). Nonetheless, since this study has shown a close link between process performance, BPM performance, and organizational performance, it seems better to refer to different performance perspectives than to differentiate between such performance types.

In future research, the comprehensiveness of the extended list of performance indicators can be empirically validated by subject-matter experts. Additionally, case studies can be conducted in which organizations apply the list as a supplement to performance measurement models in order to facilitate the selection of indicators for their specific business context. The least covered perspectives in the academic research also seem to be those that are newly emerging (namely, the perspectives related to close collaboration with suppliers, society/sustainability and process flexibility or agility), and these need more attention in future research. Another research avenue is to elaborate on the notion of a business context; for instance, by investigating what it means to have a strategic fit (Venkatraman 1989) in terms of performance measurement and which strategies (Miller and Friesen 1986; Porter 2008; Treacy and Wiersema 1993) are typically associated with which performance indicators. Additionally, the impact of environmental aspects, such as market velocity (Eisenhardt and Martin 2000), on the choice of performance indicators can be taken into account in future research.

## Conclusion

Business quotes such as “If you cannot measure it, you cannot manage it” or “What is measured improves” (P. Drucker) are sometimes criticized because not all important things seem measurable (Ryan 2014). Nonetheless, given the perceived need of managers to measure their business and the wide variety of performance indicators (i.e., ranging from quantitative to qualitative and from financial to non-financial), this structured literature review has presented the status of the research on business process performance measurement. This structured approach allowed us to detect weaknesses or inadequacies in the current literature, particularly regarding the definition and concretization of possible performance indicators. We continued by taking a holistic view of the categorization of the observed performance indicators (i.e., measures or metrics) into 11 performance perspectives based on relevant performance measurement models and established process performance dimensions.

The identified performance indicators within the 11 perspectives constitute an extended list from which practitioners and researchers can select appropriate indicators depending on their needs. In total, the structured literature review resulted in 140 possible performance indicators: 87 indicators with operationalization, 48 additional indicators that need further concretization, and 5 refinements based on other Balanced Scorecard (BSC) variants. As such, the 11 performance perspectives with related indicators can be considered a conceptual framework that was derived from the current process literature and theoretically validated by established measurement approaches in organization management.

Future research can empirically validate the conceptual framework by involving subject-matter experts to assess the comprehensiveness of the extended list and refine the missing concretizations, and by undertaking case studies in which the extended list can be applied by specific organizations. Other research avenues exist to investigate the link between actual process performance and expected process performance (as measured in maturity models) or the impact of certain strategic or environmental aspects on the choice of specific performance indicators. Such findings are needed to supplement and enrich existing performance measurement systems.

## Appendix 1

See Table 7.

**Table 7:** The final list of sampled papers (N = 76)

1	Huang SY, Lee CH, Chiu AA, Yen DC (2015) How business process reengineering affects information technology investment and employee performance under different performance measurement. Inf Syst Front 17(5):1133–1144. doi: 10.1007/s10796-014-9487-4
2	Padua, SID, Jabbour CJC (2015) Promotion and evolution of sustainability performance measurement systems from a perspective of business process management: From a literature review to a pentagonal proposal. Bus Process Manag J 21(2):403–418. doi:10.1108/BPMJ-10-2013-0139
3	Rinaldi M, Montanari R, Bottani E (2015) Improving the efficiency of public administrations through business process reengineering and simulation: A case study. Bus Process Manag J 21(2):419–462. doi:10.1108/BPMJ-06-2014-0054
4	Camara MS, Ducq Y, Dupas R (2014) A methodology for the evaluation of interoperability improvements in inter-enterprises collaboration based on causal performance measurement models. Int J Comput Integr Manuf 27(2):103–119
5	Lehnert M, Linhart A, Röglinger M (2014) Chopping down trees versus sharpening the axe—Balancing the development of BPM capabilities with process improvement. In: Sadiq S, Soffer P, Völzer H (Eds) BPM 2014. LNCS 8659. Springer, Switzerland, pp 151–167
6	del-Rio-Ortega A, Resinas M, Cabanillas C, Ruiz-Cortes A (2013) On the definition and design-time analysis of process performance indicators. Inf Syst 38(4): 470–490
7	Balaban N, Belic K, Gudelj M (2011) Business process performance management: theoretical and methodological approach and implementation. Manag Inf Syst 6(4):003–009
8	Glykas M (2013) Fuzzy cognitive strategic maps in business process performance measurement. Expert Syst Appl 40(1):1–14. doi:10.1016/j.eswa.2012.01.078
9	Hernaus T, Bach MP, Bosilj-Vuksic V (2012) Influence of strategic approach to BPM on financial and non-financial performance. Balt J Manag 7(4):376–396. doi:10.1108/17465261211272148
10	Akyuz GA, Erkan TE (2010) Supply chain performance measurement : a literature review. Int J Prod Res 48(17):5137–5155. doi:10.1080/00207540903089536
11	Han KH, Choi SH, Kang JG, Lee G (2010) Performance-centric business activity monitoring framework for continuous process improvement. AIKED Proceedings of WSEAS, pp 40–45. Available via http://dl.acm.org/citation.cfm?id=1808045. Accessed Apr 2016
12	Han KH, Kang JG, Song M (2009) Two-stage process analysis using the process-based performance measurement framework and business process simulation. Expert Syst Appl 36(3):7080–7086. doi:10.1016/j.eswa.2008.08.035
13	Cheng MY, Tsai HC, Lai YY (2009) Construction management process reengineering performance measurements. Autom Constr 18(2):183–193. doi:10.1016/j.autcon.2008.07.005
14	Alfaro JJ, Rodriguez–Rodriguez R, Verdecho MJ, Ortiz, A (2009) Business process interoperability and collaborative performance measurement. Int J Comput Integr Manuf 22(9):877–889. doi:10.1080/09511920902866112
15	Pakseresht M, Seyyedi MA, Zade MM, Gardesh H (2009) Business process measurement model based on the fuzzy multi agent systems. AIKED Proceedings of WSEAS, pp 501–506
16	Bosilj-Vuksic V, Milanovic L, Skrinjar R, Indihar-Stemberger M (2008) Organizational performance measures for business process management: A performance measurement guideline. Tenth International Conference on Computer Modeling and Simulation (UKSIM Proceedings), pp 94–99. doi:10.1109/UKSIM.2008.114
17	Wetzstein B, Ma Z, Leymann F (2008) Towards measuring key performance indicators of semantic business processes. In: Abramowicz W, Fensel D (Eds) BIS 2008, LNBIP vol 7. Springer, Berlin Heidelberg, pp 227–238. doi:10.1007/978-3-540-79396-0_20
18	Glavan LM (2012) Understanding process performance measurement systems. Bus Sys. Res J 2(2):25–38. doi:10.2478/v10305-012-0014-0
19	vom Brocke J (2007) Service portfolio measurement: evaluating financial performance of service-oriented business processes. Int J Web Serv Res 4(2):1–33
20	Korherr B, List B (2007a) Extending the EPC with performance measures. ACM Symposium on Applied Computing, pp 1265–1266
21	Korherr B, List B (2007b) Extending the EPC and the BPMN with business process goals and performance measures. ICEIS Proceedings, pp 287–294
22	Herzog NV, Polajnar A, Pizmoht P (2006) Performance measurement in business process re-engineering. J Mech Eng 52(4):210–224
23	Korherr B, List B (2006) Extending the UML 2 activity diagram with business process goals and performance measures and the mapping to BPEL. In: Roddick JF et al. (Eds) ER Workshops 2006. LNCS, vol 4231. Springer, Berlin Heidelberg, pp 7–18. doi:10.1007/11908883_4
24	Lenz K, Mevius M, Oberweis A (2005) Process-oriented business performance management with Petri nets. IEEE Proceedings, pp 89–92
25	Kuwaiti ME (2004) Performance measurement process: definition and ownership. International Journal of Operations & Production Management, 24(1):55–78
26	Kutucuoglu KY, Hamali J, Sharp JM, Irani Z (2002) Enabling BPR in maintenance through a performance measurement system framework. Int J Oper Prod Manag 14(1): 33–52. doi:10.1023/A:1013870802492
27	Jagdev H, Bradley P, Molloy O (1997) A QFD based performance measurement tool. Comput Ind 33(2–3):357–366. doi:10.1016/S0166-3615(97)00041-9
28	Bititci US, Carrie AS, McDevitt L (1997) Performance management: A business process view. IFIP WG 5.7 Proceedings, pp 284–297
29	del-Rio-Ortega A, Cabanillas C, Resinas M, Ruiz-Cortes A (2013) PPINOT tool suite: a performance management solution for process-oriented organisations. In: Basu S et al. (Eds) ICSOC Proceedings. LNCS, vol 8274. Springer, Berlin Heidelberg, pp 675–678. doi:10.1007/978-3-642-45005-1_58
30	Mirsu DB (2013) Monitoring help desk process using KPI. In: Balas VE et al. (Eds) Soft Comput Appl 195:637–647
31	Koetter F, Kochanowski M (2012) Goal-oriented model-driven business process monitoring using ProGoalML. In: Abramowicz W et al. (Eds) BIS 2012. LNBIP, vol 117. Springer, Berlin Heidelberg, pp 72–83. doi:10.1007/978-3-642-30359-3_7
32	del-Rio-Ortega A, Resinas M, Duran A, Ruiz-Cortes A (2012) Defining process performance indicators by using templates and patterns. In: Barros A, Gal A, Kindler E (Eds) BPM 2012. LNCS, vol 7481. Springer, Berlin Heidelberg, pp 223–228. doi:10.1007/978-3-642-32885-5_18
33	Arigliano F, Bianchini D, Cappiello C, Corallo A, Ceravolo P, Damiani E, De Antonellis V, Pernici B, Plebani P, Storelli D, Vicari C (2012) Monitoring business processes in the networked enterprise. In: Aberer K, Damiani E, Dillon T (Eds) SIMPDA 2011. LNBIP, vol 116. Springer, Berlin Heidelberg, pp 21–38
34	Wetzstein B, Leitner P, Rosenberg F, Dustdar S, Leymann F (2011) Identifying influential factors of business process performance using dependency analysis. Enterp Inf Syst 5(1):79–98. doi:10.1080/17517575.2010.493956
35	Shamsaei A, Pourshahid A, Amyot D (2011) Business process compliance tracking using key performance indicators. In: zur Muehlen M, Su J (Eds) BPM 2010 Workshops. LNBIP, vol 66. Springer, Berlin Heidelberg, pp 73–84
36	del-Rio-Ortega A, Resinas M, Ruiz-Cortes A (2010) Defining process performance indicators: An ontological approach. In: Meersman R et al. (Eds) OTM 2010, Part 1. LNCS, vol 6426. Springer, Berlin Heidelberg, pp 555–572
37	Pourshahid A, Amyot D, Peyton L, Ghanavati S, Chen P, Weiss M, Forster A J (2009) Business process management with the user requirements notation. Electron Commer Res 9(4):269–316. doi:10.1007/s10660-009-9039-z
38	Wetzstein B, Leitner P, Rosenberg F, Brandic I, Dustdar S, Leymann F (2009) Monitoring and analyzing influential factors of business process performance. IEEE EDOC Proceedings, pp 141–150. doi:10.1109/EDOC.2009.18
39	Liu B, Fan Y, Huang S (2008) A service-oriented business performance evaluation model and the performance-aware service selection method. Concurr Comput Pract Exp 20(15):1821–1836
40	Longo A, Motta G (2006) Design processes for sustainable performances: a model and a method. In: Bussler C et al. (Eds) BPM 2005 Workshops. LNCS, vol 3812. Springer, Berlin Heidelberg, pp 399–407
41	Zakarian A, Wickett P, Siradeghyan Y (2006) Quantitative model for evaluating the quality of an automotive business process. Int J Prod Res 44(6):1055–1074. doi:10.1080/00207540500371949
42	Wieland U, Fischer M, Pfitzner M, Hilbert A (2015) Process performance measurement system—towards a customer-oriented solution. Bus Process Manag J 21(2):312–331. doi:10.1108/BPMJ-04-2014-0032
43	Vernadat F, Shah L, Etienne A, Siadat A (2013) VR-PMS: a new approach for performance measurement and management of industrial systems. Int J Prod Res 51(23–24):7420–7438
44	Zutshi A, Grilo A, Jardim-Goncalves R (2012) The business interoperability quotient measurement model. Comput Ind 63(5):389–404. doi:10.1016/j.compind.2012.01.002
45	Ciemleja G, Lace N (2011) The model of sustainable performance of small and medium-sized enterprise. Eng Econ 22(5):501–509. doi: 10.5755/j01.ee.22.5.968
46	Chimhamhiwa D, van der Molen P, Mutanga O, Rugege D (2009) Towards a framework for measuring end to end performance of land administration business processes—A case study. Comput Environ Urban Syst 33(4):293–301. doi: 10.1016/j.compenvurbsys.2009.04.001
47	Albayrak CA, Gadatsch A, Olufs D (2009) Life cycle model for IT performance measurement: a reference model for small and medium enterprises (SME). In: Dhillon G, Stahl BC, Baskerville R (Eds) CreativeSME 2009. IFIP AICT, vol 301, pp 180–191. Available via http://link.springer.com/chapter/10.1007/978-3-642-02388-0_13
48	Hinrichs N, Barke E (2008) Applying performance management on semiconductor design processes. IEEE IEEM Proceedings, pp 278–281. doi:10.1109/IEEM.2008.4737874
49	Adams TM, Danijarsa M, Martinelli T, Stanuch G, Vonderohe A (2003) Performance measures for winter operations. Transp Res Rec J Transp Res Board 1824:87–97. doi: 10.3141/1824-10
50	Kueng P (2000) Process performance measurement system: a tool to support process-based organizations. Total Qual Manag 11(1):67–85. doi: 10.1080/0954412007035
51	Kueng P, Krahn AJW (1999) Process performance measurement system: some early experiences. J Scien Ind Res 58(3–4):149–159
52	Walsh P (1996) Finding key performance drivers: some new tools. Total Quality Management. 7(5):509–519. doi: 10.1080/09544129610612
53	Fogarty DW (1992) Work in process: performance measures. Int J Prod Econ 26(1–3):169–172. doi:10.1016/0925-5273(92)90059-G
54	Gunasekaran A, Patel C, McGaughey RE (2004) A framework for supply chain performance measurement. Int J Prod Econ 87(3):333–347. doi:10.1016/j.ijpe.2003.08.003
55	Gunasekaran A, Kobu B (2007) Performance measures and metrics in logistics and supply chain management : a review of recent literature (1995—2004) for research and applications. Int J Prod Res 45(12):37–41. doi:10.1080/00207540600806513
56	Wang CH, Lu IY, Chen CB (2010) Integrating hierarchical balanced scorecard with non-additive fuzzy integral for evaluating high technology firm performance. Int J Prod Econ 128(1):413–426. doi:10.1016/j.ijpe.2010.07.042
57	Wu HY (2012) Constructing a strategy map for banking institutions with key performance indicators of the balanced scorecard. Eval Program Plann 35(3):303–320. doi:10.1016/j.evalprogplan.2011.11.009
58	Martinsons M, Davison R, Tse D (1999) The balanced scorecard: a foundation for the strategic management of information systems. Decis Support Syst 25(1):71–88. doi: 10.1016/S0167-9236(98)00086-4
59	Grigoroudis E, Orfanoudaki E, Zopounidis C (2012) Strategic performance measurement in a healthcare organisation: A multiple criteria approach based on balanced scorecard. Omega 40(1):104–119. doi:10.1016/j.omega.2011.04.001
60	Bhagwat R, Sharma MK (2007) Performance measurement of supply chain management: a balanced scorecard approach. Comput Ind Eng 53(1):43–62. doi:10.1016/j.cie.2007.04.001
61	Al-Mashari M, Al-Mudimigh A, Zairi M (2003) Enterprise resource planning: a taxonomy of critical factors. Eur J Oper Res 146(2):52–364. doi:10.1016/S0377-2217(02)00554-4
62	Jalali NSG, Aliahmadi AR, Jafari EM (2011) Designing a mixed performance measurement system for environmental supply chain management using evolutionary game theory and balanced scorecard: a case study of an auto industry supply chain. Resour Conserv Recycl 55(6):593–603. doi: 10.1016/j.resconrec.2010.10.008
63	Huang HC (2009) Designing a knowledge-based system for strategic planning: a balanced scorecard perspective. Expert Syst Appl 36(1):209–218. doi:10.1016/j.eswa.2007.09.046
64	Bosilj-Vuksic V, Glavan LM, Susa D (2015) The role of process performance measurement in BPM adoption outcomes in Croatia. Econ Bus Rev 17(1):117–143. Available via http://eserv.uum.edu.my/docview/1692430846?accountid=42599
65	Jahankhani H, Ekeigwe JI (2005) Adaptation of the balanced scorecard model to the IT functions. IEEE ICITA Proceedings, pp 784–787. doi:10.1109/ICITA.2005.52
66	Spremic M, Zmirak Z, Kraljevic K (2008) IT and business process performance management: case study of ITIL implementation in finance service industry. ITI Proceedings, pp 243–250. doi:10.1109/ITI.2008.4588415
67	Li S, Zhu H (2008) Generalized stochastic workflow net-based quantitative analysis of business process performance. IEEE ICINFA Proceedings, pp 1040–1044. doi:10.1109/ICINFA.2008.4608152
68	Cardoso ECS (2013) Towards a methodology for goal-oriented enterprise management. IEEE EDOC Proceedings, pp 94–103. doi:10.1109/EDOCW.2013.17
69	Tung A, Baird K, Schoch HP (2011) Factors influencing the effectiveness of performance measurement systems. Int J Oper Prod Manag 31(12):1287–1310. doi:10.1108/01443571111187457
70	Koetter F, Kochanowski M (2015) A model-driven approach for event-based business process monitoring. Inf Syst E-bus Manag 13(1):5–36. doi:10.1007/s10257-014-0233-8
71	Banker RD, Chang H, Janakiraman SN, Konstans C (2004) A balanced scorecard analysis of performance metrics. Eur J Oper Res 154(2):423–436. doi:10.1016/S0377-2217(03)00179-6
72	Peng Y, Zhou L (2011) A performance measurement system based on BSC. In: Zhu M (Ed) ICCIC 2011, Part V. CCIS, vol 235. Springer, Berlin Heidelberg, pp 309–315
73	van Heck G, van den Berg J, Davarynejad M, van Duin R, Roskott B (2010) Improving inventory management performance using a process-oriented measurement framework. In: Quintela Varajao JE et al. (Eds) CENTERIS 2010, Part I. CCIS, vol 109. Springer, Berlin Heidelberg, pp 279–288
74	Caputo E, Corallo A, Damiani E, Passiante G (2010) KPI modeling in MDA Perspective. In: Meersman R et al. (Eds) OTM 2010 Workshops. LNCS, vol 6428. Springer, Berlin Heidelberg, pp 384–393. doi:10.1007/978-3-642-16961-8_59
75	Behrouzi F, Shaharoun AM, Ma’aram A (2014) Applications of the balanced scorecard for strategic management and performance measurement in the health sector. Aust Heal Rev 38(2):208–217. doi:10.1071/AH13170
76	Skrinjar R, Indihar-Stemberger M (2009) Improving organizational performance by raising the level of business process orientation maturity: empirical test and case study. In: Barry C et al. (Eds) Information Systems Development: Challenges in Practice, Theory and Education. Springer, Heidelberg, pp 723–740. doi:10.1007/978-0-387-78578-3_11

## Appendix 2: The mapping of the structured literature review

The mapping details per sampled paper can be found here.

https://drive.google.com/file/d/0B_2VpjwsRLrlRHhfRHJ4ZFBWdEE/view?usp=sharing.

## Appendix 3

See Table 8.

**Table 8:** The list of performance indicators with operationalization

Perspectives	Indicators/measures/metrics	Operationalization	Papers
1/Financial performance			
	Sales performance	[Achieved total sales]/[planned sales] * 100	7
	Inventory turnover	[Annual total sales]/[average inventory] * 100	59
	Market share	% of growth in the last years [Sales volumes of products and services]/[total market demands] * 100	16, 57
	Earnings per share (EPS)	[After-tax net earnings − preferred share dividends]/[weighted average nr of shares outstanding]	57
	Average order value	[Aggregated monthly sales]/[monthly nr of orders]	7
	Order growth	[Number of orders in the current month]/[total nr of orders]	7
	Revenue growth	[Revenue from new sources]/[total revenue] * 100	16
	Operating revenue	Sales revenues	57
	Return on investment (ROI)	[After-tax profit or loss]/[total costs] [Revenue − cost]/[cost]	57, 55
	Return on assets (ROA)	[After-tax profit or loss]/[average total assets]	57, 16
	Circulation of assets	[Operating revenues]/[assets] * 100	59
	Current ratio	[Current assets]/[current liabilities] * 100	59
	Net profit margin	[After-tax profit or loss]/[total operating revenues] [Total operating revenues − operating expenses − non-operating expenses]/[total operating revenues]	16, 57, 59
	Profit per customer	[After-tax earnings]/[total nr of online, offline or all customers]	57
	Management efficiency	[Operating expenses]/[operating revenues] * 100	59
	Debt ratio, leverage level	[Debts]/[assets]	57, 59
2/Customer performance			
2.1/Customer performance			
	Customer complaints, return rate	Nr of complaints, criticisms or notifications due to dissatisfaction about or non-compliance of orders, products and services Nr or % of orders returned, rework or services to be redone (e.g., incorrect deliveries, incorrect documentation)	27, 30, 37, 40, 51, 57, 59
	Perceived customer satisfaction	Qualitative scale on general satisfaction (e.g., Likert), possibly indexed as the weighted sum of judgements on satisfaction dimensions (e.g., satisfaction with products and services, perceived value, satisfying end-user needs, being the preferred suppliers for products or services, responsiveness, appearance, cleanliness, comfort, friendliness, communication, courtesy, competence, availability, security)	5, 16, 22, 40, 46, 11, 55 57, 59, 58, 60
	Perceived customer easiness	Qualitative scale (e.g., Likert) on the degree of easiness to find information and regulations, to fill out applications, and to understand the presentation of bureaucratic language	40
	Customer retention	Nr of returning customers	57
	Customer growth	Nr of new customers	57
	Customer query time, resolution time, response time	Average time between issuing and addressing a customer problem or inquiry for information	30, 40, 46, 58, 59, 60
	Customer waiting time	[Time for information about a product or service] + [time for following status updates] + [time for receiving the product or service] Max nr of customers in the queue or waiting room [Handled requests]/[total requests]	3, 40, 52, 59
	Punctuality, delivery reliability	[Late deliveries or requests]/[total nr of deliveries or requests] % of On-time deliveries according to the planning or schedule	16, 18, 26, 27, 40, 51, 55, 60, 73
	Payment reliability	[Nr of collected orders paid within due date]/[total nr of orders] * 100	7
	Information access cost, information availability	Information provided/not provided Time spent in asking for information about a product or service (in days) Time required to get updated about the status of a product or service Cost of information (euro)	40
	Customer cost	Product cost or the cost of using a service (euro)	40
2.2/Supplier performance			
	External delays	Nr of delayed deliveries due to outage or delays of third-party suppliers	26, 73
	External mistakes	% of Incorrect orders received	27
	Transfers, partnerships	% of Cases transferred to a partner	59
2.3/Society performance			
	Perceived society satisfaction	Qualitative scale on general satisfaction (e.g., Likert), possibly indexed as the weighted sum of judgements on satisfaction dimensions % of Society satisfied with the organization’s outcomes	46
	Societal responsibility, sustainability, ecology, green	Number of realized ecology measures (e.g., waste, carbon dioxide, energy, water) Quantity of carbon dioxide emitted per man month	51
3/Business process performance			
3.1/General process performance			
	Process complexity	Number of elementary operations to complete the task	40
	General process information	Nr of orders received or shipped per time unit Nr of incoming calls per time unit Nr of process instances	6, 27, 52
	Order execution	[Nr of executed orders]/[total nr of orders] * 100	7
	Perceived sales performance	Qualitative scale (e.g., Likert) on the successful promotion of both efficiency and effectiveness of sales	57
	Perceived management performance	Qualitative scale (e.g., Likert) on the improvement of effectiveness, efficiency, and quality of each objective and routine tasks	57
	Surplus inventory	% of current assets Value of surplus inventory (e.g., pharmaceutical material) to total assets ratio	59
	Occupancy rate	Average % occupancy, e.g., of hospital beds	59
**3.2/**Time-related process performance			
	Throughput	Nr of processed requests/time unit	46
	Process duration, efficiency	[Σ(finish date − start date) of all finished business objects]/[number of all finished business objects]	17
	Process cycle time, order cycle time, process duration, average lifetime, completion time, process lead time	Time for handling a process instance end-to-end Aggregated time of all activities associated with a process (per instance) [Application submission time] − [application response time]	5, 6, 11, 37, 40, 43, 46, 60, 73
	Average sub-process turnaround time, task time, activity time	[Sub-process start time] − [Sub-process finish time]	6, 37, 40, 52, 60
	Processing time	Time that actual work is performed on a request	46
	Average order execution time, order fulfillment time, order lead time	[Σ(Dispatch time − creation time)]/[total number of orders] [order entry time] + [order planning time] + [order sourcing, assembly and follow-up time] + [finished goods delivery time]	7, 46, 60, 73
	Average order collection time	[Σ(Collection time − creation time)]/[number of collected orders]	7
	Average order loading time	[Σ(Final distribution time − distribution creation time)]/[number of loaded orders]	7
	Process waiting time, set-up time	Average time lag between sub-processes, when a process instance is waiting for further processing Time between the arrival of a request and the start of work on it (=time spent on hold) Average waiting time for all products and services	3, 5, 20, 37, 46, 52
	Manufacturing cycle efficiency	[setup time + (nr of parts * operation time)]/[manufacturing lead time]	53
	Manufacturing lead time	[setup time + (nr of parts * operation time) + queue time + wait time + movement time]	18, 53, 55
	Value added efficiency	[Operation time]/[manufacturing lead time]	53
3.3/Cost-related process performance			
	Activity cost	Cost of carrying out an activity	46
	Process cost, cost of quality, cost of producing, customer order fulfilment cost	Sum of all activity costs associated with a process (per instance)	5, 11, 16, 18, 20, 22, 26, 27, 40, 43, 46
	Unit cost	Nr of employees (headcount) per application, product or service	40
	Information sharing cost	[Time for system data entry] + [time for system delivery output]	40
3.4/Process performance related to internal quality			
	Quality of internal outputs, external versus internal quality, error prevention	% of instance documents processed free of error Number of mistakes [Nr of tasks with errors]/[Total nr of tasks per process] Nr of syntactic errors Nr of repeated problems Presence of non-technical anomaly management (yes/no)	5, 16, 18, 20, 22, 37, 40, 43, 46, 55, 60, 66
	Deadline adherence, schedule compliance, due date performance effectiveness, responsiveness	% of Activity cycle times realized according to the planning or schedule [Number of finished business objects on time]/[number of all finished business objects] * 100	16, 17, 18, 26, 43
	Process yield	Multiply the yield per process steps, e.g., (1 − scrap parts/total parts)^step 1^ * (1 − scrap parts/total parts)^step 2^	43
	Rework time, transaction efficiency	Time to redo work for an incident that was solved partially or totally incorrect the first time Average time spent on solving problems occurring during transactions	30, 43, 57
	Integration capability	Time to access and integrate information	40
3.5/Process performance related to flexibility			
	Special requests	Nr of special cases or requests	40
4/“Learning and growth”-performance			
4.1/(Digital) innovation performance			
	Degree of digitalization	% Reduction in processing time due to computerization [Nr of process steps replaced by computer systems]/[Total nr of steps in the entire process] Nr of digital products or services	40, 46, 71
	Degree of rationalization	% of Procedures and processes systemized by documentation, computer software, etc.	57
	Time for training on the procedure	Measured in hours	40
	Novelty in output	Nr of new product or service items	57
	Customer response	Nr of suggestions provided by customers about products and services	57
	Third-party collaboration	Nr of innovation projects conducted with external parties	59
	Innovation projects	Nr of innovations proposed per quarter year Nr of innovations implemented per quarter year	51
	IS development efficiency	Nr of change requests (+per type of change or per project) Time spent to repair bugs and finetune new applications Time required to develop a standard-sized new application % of Application programming with re-used code	6, 58, 66
	Relative IT/IS budget	[Total IT/IS budget]/[Total revenue of the organization] * 100	58
	Budget for buying IT/IS	[Budget of IT/IS bought]/[Total budget of the organization] * 100	59
	Budget for IS training	[IS training budget]/[overall IS budget] * 100	58
	Budget for IS research	[IS research budget]/[overall IS budget] * 100	58
	Perceived management competence	Qualitative scale (e.g., Likert) on the improvement in project management, organizational capability, and management by objectives (MBO)	57
	Perceived relationship between IT management and top management	Qualitative scale (e.g., Likert) on the perceived relationship, time spent in meetings between IT and top management, and satisfaction of top management with the reporting on how emerging technologies may be applicable to the organization	58
4.2/Employee performance			
	Perceived employee satisfaction	Qualitative scale on general satisfaction (e.g., Likert), possibly indexed as the weighted sum of judgements on satisfaction dimensions Qualitative scale (e.g., Likert) on satisfaction about hardware and software provided by the organization	16, 43, 11, 57, 58, 59
	Average employee saturation, resource utilization for process work	[Time spent daily on working activities]/[total working time] * 100 [Work time]/[available time] % of operational time that a resource is busy	3, 40, 46
	Resource utilization for (digital innovation)	IS expenses per employee % of Resources devoted to IS development % of Resources devoted to strategic projects	58
	Process users	Nr of employees involved in a process	37
	Working time	Actual time a business process instance is being executed by a role	20
	Workload	Nr of products or services handled per employee	71
	Staff turnover	% of Employees discontinuing to work and replaced, compared to the previous year	16, 57, 58
	Employee retention, employee stability	% of Employees continuing to work in the organization, compared to the previous year	16, 57, 58, 59
	Employee absenteeism	[Total days of absence]/[total working days for all staff] * 100	59
	Motivation of employees	Average number of overtime hours per employee	16
	Professional training, promotion and personal development	% of Employees trained % of Employees participated in a training program per year Nr of professional certifications or training programs per employee	57, 59, 22
	Professional conferences	% of Employees participating in conferences	59

## Appendix 4

See Table 9.

**Table 9:** Additional list of performance indicators without operationalization

Perspectives	Performance indicators/measures/metrics	Papers
1/Financial performance		
	Selling price	18, 55
	Cash flow	22
2/Customer performance		
2.1/Customer performance		
	Customer relationship management, direct customer cooperation, efficiency of customer cooperation, establishing and maintaining relationships with the user community	11, 22, 58
	Warranty cost	55
	Delivery cost	27
	Delivery frequency	18, 60, 73
2.2/Supplier performance		
	Efficiency of cooperation with vendors, buyer–supplier partnership level, degree of collaboration and mutual assistance, nr of supplier contracts	11, 60, 73
	Information carrying costs, level and degree of information sharing	60
	Supplier rejection rate	60
	Buyer-vendor cost saving initiatives	60
	Delivery frequency	60
	Supplier ability to respond to quality problems	60
	Supplier’s booking in procedures	60
	Supplier lead time against industry norms	60
3/Business process performance		
3.3/Cost-related process performance		
	Cost of risks	58
	Cost per operating hour, running cost	18, 60
	Material cost	22
	Service cost	18, 22
	Inventory cost (e.g., incoming stock level, work-in-progress, scrap value, finished goods in transit)	22, 55, 60
	Overhead cost	55
	Obsolescence cost	55
	Transportation cost	55
	Maintenance cost	26
3.4/Process performance related to internal quality		
	Conformance to specifications	55
	Compliance with regulation	18, 43, 55
	Verification mismatches	73
	Forecasting accuracy, accuracy of scheduling	55, 60, 73
3.5/Process performance related to flexibility		
	Process flexibility	22, 58
	General flexibility	5, 22, 40
	Product or service variety	55
	Range of products or services	60
	Modification of products or services, volume mix, resource mix	18, 22, 55
	Flexibility of service systems to meet particular customer needs	60
	Effectiveness of delivery invoice methods	60
	Payment methods	52
	Order entry methods	60
	Responsiveness to urgent deliveries	60
4/“Learning and growth”-performance		
4.1/(Digital) innovation performance		
	R&D performance, investment in R&D and innovations	11, 16
	New product or service development costs	22
	Knowledge base	16
4.2/Employee performance		
	Productivity	11, 22, 40
	Labor efficiency	55
	Labor cost	22
	Employee availability	22, 26, 40, 52
	Expertise with specific existing technologies	58
	Expertise with specific emerging technologies	58
	% of multi-skilled workforce	26
	Age distribution of IS staff	58

## Abbreviations

BH: behavioral science
BPM: business process management
BSC: balanced scorecard
DS: design-science
RQ: research question
SLR: structured literature review
TO: keyword in topic
TI: keyword in title
